# Supplementary material for: Causal Relationship between Aging and Anorexia Nervosa: A White-Matter-Microstructure-Mediated Mendelian Randomization Analysis
Source: Biomedicines. 2024 Aug 16;12(8):1874. doi: 10.3390/biomedicines12081874 (PMC11351342; doi:10.3390/biomedicines12081874)
Supplement: Supplementary file 1 [file biomedicines-12-01874-s001.zip › Supplementary Figure/Figure S8/Leaveoneout plot of MR analysis for TL to ubm-b-1537.pdf]

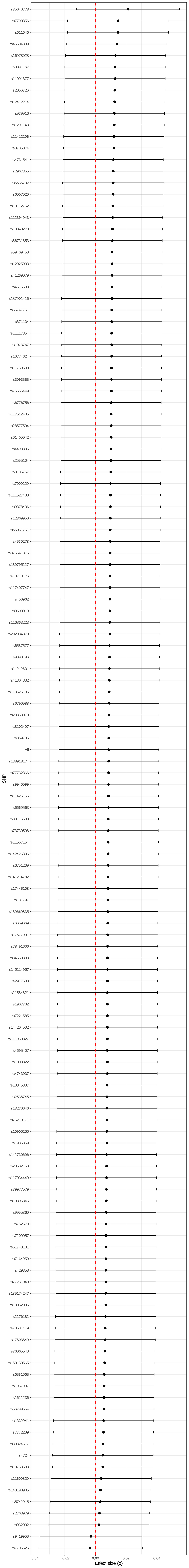
SNP
rs35640778
rs7790856
rs611646
rs45604339
rs16978028
rs3891167
rs11991877
rs2056726
rs12412214
rs939916
rs1291143
rs11412296
rs3785074
rs4731541
rs2967355
rs6536702
rs6007020
rs10112752
rs112394943
rs10840270
rs66731853
rs59409453
rs12925933
rs41269079
rs4616688
rs137901416
rs55747751
rs871134
rs11117354
rs1023767
rs10774624
rs11769630
rs3093888
rs76666449
rs6776756
rs117512405
rs28577594
rs61405042
rs4498805
rs2555104
rs8105767
rs7099229
rs111527438
rs9878436
rs12369950
rs56061761
rs4530278
rs376641875
rs139795227
rs10773176
rs117407747
rs450962
rs9600019
rs116863223
rs202034370
rs6587577
rs9398196
rs11212631
rs41304832
rs113525195
rs6790988
rs28363070
rs8102497
rs869785
All
rs188918174
rs77732866
rs9940099
rs11426156
rs6669563
rs80116508
rs73730598
rs11557154
rs142426306
rs6751209
rs141214782
rs17445108
rs131797
rs139669835
rs6659669
rs17677991
rs78491606
rs34550383
rs145114957
rs2977608
rs11584821
rs1907702
rs7221585
rs144204502
rs111950327
rs4695407
rs1003322
rs4743037
rs10845387
rs2538745
rs13230646
rs76219171
rs10905255
rs1985369
rs142730696
rs28502153
rs117034449
rs79977579
rs10805346
rs9955360
rs762679
rs7209057
rs61748181
rs7164950
rs429358
rs77231040
rs185174247
rs13062095
rs2276182
rs73581419
rs17803849
rs76065543
rs150150565
rs6881568
rs1957937
rs1611236
rs56799554
rs1332941
rs7772289
rs80324517
rs4724
rs10768683
rs11699829
rs143190905
rs5742915
rs2763979
rs932002
rs9419958
rs7705526
−0.04
−0.02
0.00
0.02
0.04
Effect size (b)
